# National trends and projection of chronic kidney disease incidence according to etiology from 1990 to 2030 in Iran: a Bayesian age-period-cohort modeling study

**DOI:** 10.4178/epih.e2023027

**Published:** 2023-02-17

**Authors:** Fatemeh Shahbazi, Amin Doosti-Irani, Alireza Soltanian, Jalal Poorolajal

**Affiliations:** 1 Department of Epidemiology, School of Public Health, Hamadan University of Medical Sciences, Hamadan, Iran; 2 Students Research Committee, Hamadan University of Medical Sciences, Hamadan, Iran; 3 Health Sciences Research Center, Health Sciences & Technology Research Institute, Hamadan University of Medical Sciences, Hamadan, Iran; 4 Department of Biostatistics, School of Public Health, Hamadan University of Medical Sciences, Hamadan, Iran; 5 Modeling of Noncommunicable Diseases Research Center, Hamadan University of Medical Sciences, Hamadan, Iran

**Keywords:** Chronic renal insufficiency, Incidence, Prediction, Forecast, Trend

## Abstract

**OBJECTIVES:**

Chronic kidney disease (CKD) is a major public health problem worldwide. Predicting CKD incidence rates and case numbers at the national and global levels is vital for planning CKD prevention programs.

**METHODS:**

Data on CKD incidence rates and case numbers in Iran from 1990 to 2019 were extracted from the Global Burden of Disease online database. The average annual percentage change was computed to determine the temporal trends in CKD age-standardized incidence rates from 1990 to 2019. A Bayesian age-period-cohort model was used to predict the CKD incidence rate and case numbers through 2030.

**RESULTS:**

Nationally, CKD cases increased from 97,300 in 1990 to 315,500 in 2019. The age-specific CKD incidence rate increased from 168.52 per 100,000 to 382.98 per 100,000 during the same period. Between 2020 and 2030, the number of CKD cases is projected to rise to 423,300. The age-specific CKD incidence rate is projected to increase to 469.04 in 2030 (95% credible interval, 399.20 to 538.87). In all age groups and etiological categories, the CKD incidence rate is forecasted to increase by 2030.

**CONCLUSIONS:**

CKD case numbers and incidence rates are anticipated to increase in Iran through 2030. The high level of CKD incidence in people with diabetes mellitus, hypertension, and glomerulonephritis, as well as in older people, suggests a deficiency of attention to these populations in current prevention plans and highlights their importance in future programs for the national control of CKD.

## GRAPHICAL ABSTRACT

[Fig f6-epih-45-e2023027]

## INTRODUCTION

Chronic kidney disease (CKD) is a worldwide public health problem that reduces kidney function over a long period [1]. CKD is a non-curable chronic progressive disease with high morbidity and mortality in adults, especially those with diabetes and hypertension. CKD is associated with adverse clinical outcomes, poor quality of life, and high healthcare costs [2]. The global prevalence of CKD is about 13.4% [3], and its global mortality rate is 15.13 per 100,000 population [4]. CKD is associated with an increased risk of cardiovascular disease, hypertension, anemia, mineral bone disorder, hyperkalemia, and other complications [5-9]. By 2040, CKD is estimated to become the fifth leading cause of death globally, one of the largest projected increases of any major cause of death [10].

Multiple risk factors for developing CKD include diabetes mellitus (types 1 and 2), hypertension, glomerulonephritis, and unknown causes that present obstacles to the global control of its complications [11]. The risk factors for CKD are also rapidly changing over time. For example, between 1990 and 2019, the global prevalence of type 1 diabetes mellitus increased from 28.78 per 100,000 to 58.85 per 100,000 people, and it is forecasted to reach 61.23 per 100,000 people by 2030 [12]. Furthermore, between 2000 and 2016, there was a 5% increase in premature mortality rates from diabetes [13]. Hypertension is another risk factor for CKD. The number of adults with hypertension in 2025 is predicted to increase by about 60% to 1.56 billion [14]. Changing trends in risk factors might result in substantial alternations in CKD case numbers and incidence rates. Therefore, forecasts of these parameters are of significant interest to epidemiologists and policymakers and are of great importance in understanding and planning for the disease burden. To address this need, we used data on CKD from 1990 to 2019 at the national level to forecast the coming number of CKD patients and its incidence rate through 2030 in Iran.

## MATERIALS AND METHODS

### Study data

This ecological study analyzed secondary data. We collected annual data on CKD cases in Iran between 1990 and 2019 by sex, age (at 5-year intervals), province, and etiology (type 1 diabetes mellitus, type 2 diabetes mellitus [T2DM], hypertension, glomerulonephritis, and other causes) from the online Global Burden of Disease (GBD) query tool [12]. The general methods of the GBD study and the methods for estimating the disease burden of CKD have been detailed in previous studies [15]. We also retrieved the corresponding population data by year (1990-2019), sex, and age (at 5-year intervals) from the United Nations Department of Economics and Social Affairs Population Division [16].

### Prediction of chronic kidney disease incidence rates and case numbers, 2020-2030

**Figure 1.**
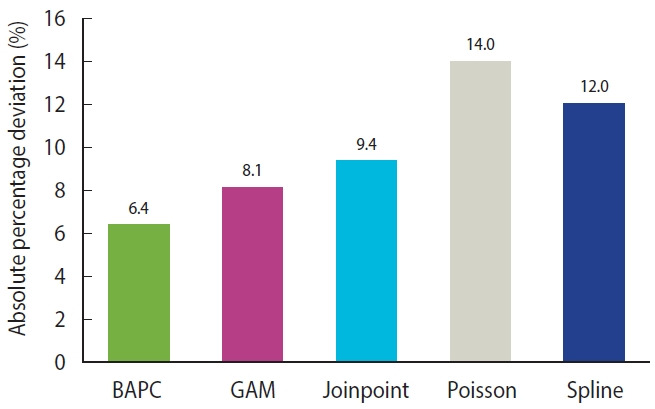
The results of model comparison based on data at the national level in Iran. BAPC, Bayesian age-period-cohort; GAM, generalized additive model.

Using CKD case data in Iran, we compared the following 5 models to select the best one with the highest predictive performance: a Bayesian age-period-cohort (BAPC) model, a generalized additive model, a smooth spline model, a joinpoint regression model, and a Poisson regression model. For this purpose, we split the dataset into 2 intervals (1990-2013 and 2014-2019). The first interval was considered a training set, and the second was a testing set of the predictive models. The best approach for determining an unknown parameter in a mathematical model, or choosing the best model among several models, is a division of the dataset into a training set and testing set. Empirical studies have shown that the best results are obtained by using 20-30% of the data for testing and the remaining 70-80% for training [17]. Therefore, we considered 80% of the dataset (1990-2013) as a training set. The CKD incidence rates and case numbers between 2015 and 2019 were predicted and compared with the actual values in the same period. The absolute percentage deviation (APD) was applied to assess model performance. The APD can be calculated as (Ŷ-Y)/Y× 100, where Ŷ and Y denote the dataset’s predicted and actual values, respectively. The APDs for all selected models are presented in Figure 1. Because of the lower APD for the BAPC model, we used it to predict CKD incidence rates and case numbers through 2030, although the values related to other models are presented in Supplementary Materials 1-8. We forecasted the case count and incidence rate of CKD for the next 11 years. There are no fixed rules for determining the duration of the forecast period. Intuitively, the longer the period, the easier it is to make an accurate forecast. For example, yearly forecasts eliminate seasonal variations. Meanwhile, a short forecasting period, such as daily forecasts, might provide a false sense of accuracy. The choice of 2030 for the endpoint of the predictions is related to the Sustainable Development Goals, which were approved by heads of state and high-level representatives of United Nations specialized agencies and civil society in September 2015 and the United Nations General Assembly, and they set the goal of reaching them by 2030. The main goal of this document is to end poverty in all its forms everywhere [18]. Accordingly, most studies in the field of public health and medicine have set the endpoint of their predictions to 2030. As another consideration, it might be sensible to level off the exponential trend assumed by the BAPC model presented here for predicting further than 10 years into the future.

We aimed to project case numbers and age-specific CKD incidence rates for 11 years in the future (2020-2030) based on the demographic and etiological findings by conducting a BAPC analysis with integrated nested Laplace approximation (INLA). This approach has been documented and validated elsewhere. Briefly, since the expectation that effects adjacent in time might be similar, a second-order random walk (RW2) model with the inverse-gamma prior distribution was used for age, period, and cohort effects. RW2 assumes an independent mean-zero normal distribution on the second differences of all time effects. This is a natural target for smoothing since the second differences in ageperiod-cohort models are identifiable. For instance, considering the age effects, the RW2 prior is given by:


$$f\left(a|k_{a}\right)\propto k_{a}^{\frac{I-2}{2}}exp\left(\frac{K_{a}}{2}\sum_{i=3}^{I}{\left(a_{i}-2a_{i-1}+a_{i-2}\right)}^{2}\right)\;=k_{a}^{\frac{I-2}{2}}exp\left(-\frac{1}{2}a^{T}Qa\right)\;Q=K_{a}\left[\begin{array}{ccccccc} 1 & 2 & 1 & & & & \\ -2 & 5 & -4 & 1 & & & \\ 1 & -4 & 6 & -4 & 1 & & \\ & \ddots & \ddots & \ddots & \ddots & \ddots & \\ & & 1 & -4 & 6 & -4 & 1\\ & & & 1 & -4 & 5 & -2\\ & & & & 1 & -2 & 1 \end{array}\right]$$

Where in the above equations, *i* denotes the age index, which ranged from 1 to 4 in this study, because we projected the CKD incidence rate and case numbers of people aged 0-84 and the age was divided into 4 groups (0-19, 20-39, 40-59, and ≥ 60 years). Moreover, $k_{a}^{-1}$ denotes the variance parameter. It should be noted that Q is rank-deficient. To complete the RW2 model specification, we used the usual conjugate hyperprior for the precision kα~gamma (α, λ). This leads to the full conditional kα|α~gamma (α+0.05 rank [Q], λ+0.5 α´ Qα), which may be directly simulated. In this study, we used the parameter default values α= 0.0005 and 1 for the age effect, β= 0.0005, 0.00005 for the period effect, and γ= 0.00005 for the cohort effects [19]. The world population in 2,000 was used to standardize the CKD incidence rates. All statistical analyses were performed using the R version 4.1.2 (R Foundation for Statistical Computing, Vienna, Austria). The BAPC model was conducted with the BAPC and INLA packages in R.

### Quantifying the chronic kidney disease incidence trends

The average annual percentage change (AAPC) was used to describe the temporal trends of CKD age-standardized incidence rates (ASRs) in 1990-2019 and 2020-2030, indicating the past and future trends, respectively. A regression line was fitted to the natural logarithm of the rates: y=α+βx+ɛ, where y is the natural logarithm of ASR and x indicates the calendar year, and the AAPC was calculated as (exponential [β]−1)× 100. The Joinpoint Regression version 4.7.0.0 (National Cancer Institute, Bethesda, MD, USA) was used to carry out the trend analysis.

### Ethics statement

This article did not involve direct human participants.

## RESULTS

### Chronic kidney disease case numbers and incidence, 1990-2019

**Figure 2.**
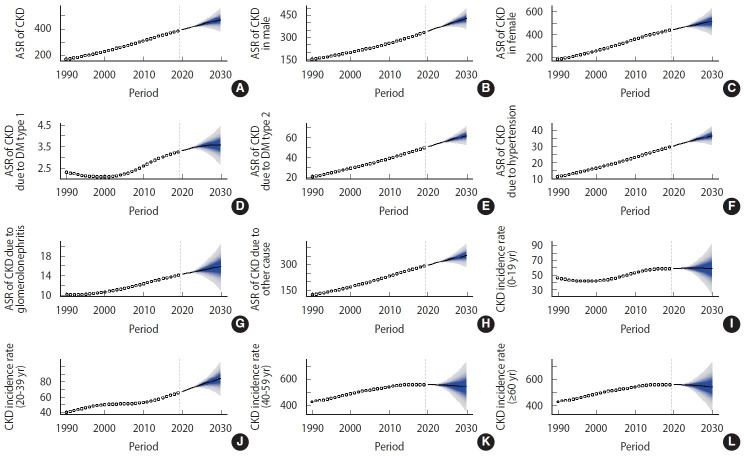
Temporal trends in the ASRs (per 100,000) of CKD between 1990 and 2019 and their projections up to 2030 at the national level in Iran (A: both sexes, B: male, C: female, D: DM type 1, E: DM type 2, F: hypertension, G: glomerulonephritis, H: other cause, I: 0-19 years, J: 20-39 years, K: 40-59 years, L: ≥60 years). The incidence rates for the 4 age groups are crude, not age-standardized. The open dots represent the observed values, and the fan shape denotes the predicted distribution between the 2.5% and 97.5% quintiles. The predicted mean value is shown as a solid line. The vertical dashed line indicates where the prediction starts. ASR, age-standardized incidence rate; CKD, chronic kidney disease; DM, diabetes mellitus.

**Figure 3.**
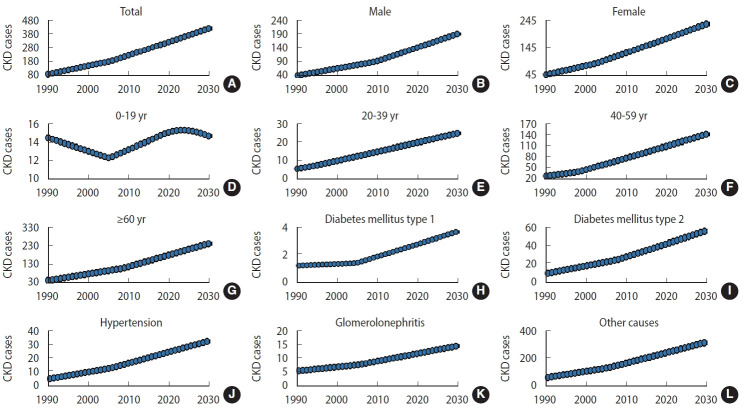
The changing trends in case numbers of chronic kidney disease (CKD), by age group, sex, and etiology between 1990 and 2017 and the predictions of case numbers through 2030 (A: total, B: male, C: female, D: 0-19 years, E: 20-39 years, F: 40-59 years, G: ≥60 years, H: diabetes mellitus type 1, I: diabetes mellitus type 2, J: hypertension, K: glomerulonephritis, L: other causes). The y-axes are on a scale of thousands.

Nationally, the new case numbers of CKD increased from 97,300 in 1990 to 315,500 in 2019, and the CKD ASR increased from 168.52 per 100,000 to 382.98 per 100,000 during the same period, with an AAPC of 2.9% (95% confidence interval [CI], 2.8 to 3.0) (Table 1, Figures 2 and 3). The incidence increased in both sexes (Table 1 and Figure 2). The incident cases of CKD in male increased from 43,700 in 1990 to 139,400 in 2019, and in female increased from 53,500 in 1990 to 176,100 in 2019. During the same period, the ASR in male and female increased from 149.38 per 100,000 to 330.61 per 100,000 and from 188.43 per 100,000 to 436.57 per 100,000, respectively. The ASR increased significantly among male, with an AAPC of 2.8% (95% CI, 2.7 to 2.9). Female experienced nearly the same increase in the ASR, with an AAPC of 2.9% (95% CI, 2.8 to 3.0). The case numbers and ASR were higher in female than in male during the study period. The CKD case numbers increased in all age groups, except people aged 0-19 years (Table 1 and Figure 3). The most pronounced increase was found in people older than 60, who exhibited an increase of more than 3.5 times between 1990 and 2019. Among the etiological factors of CKD, T2DM had the highest new case numbers and ASR. These values increased during the study period; specifically, the case number increased from 11,700 in 1990 to 41,000 in 2019, and the ASR increased from 20.02 per 100,000 in 1990 to 49.03 per 100,000 in 2019. Increasing trends were also observed for other CKD etiological factors (Table 1 and Figure 2).

### Projection analysis: chronic kidney disease case numbers and incidence, 2020-2030

**Figure 4.**
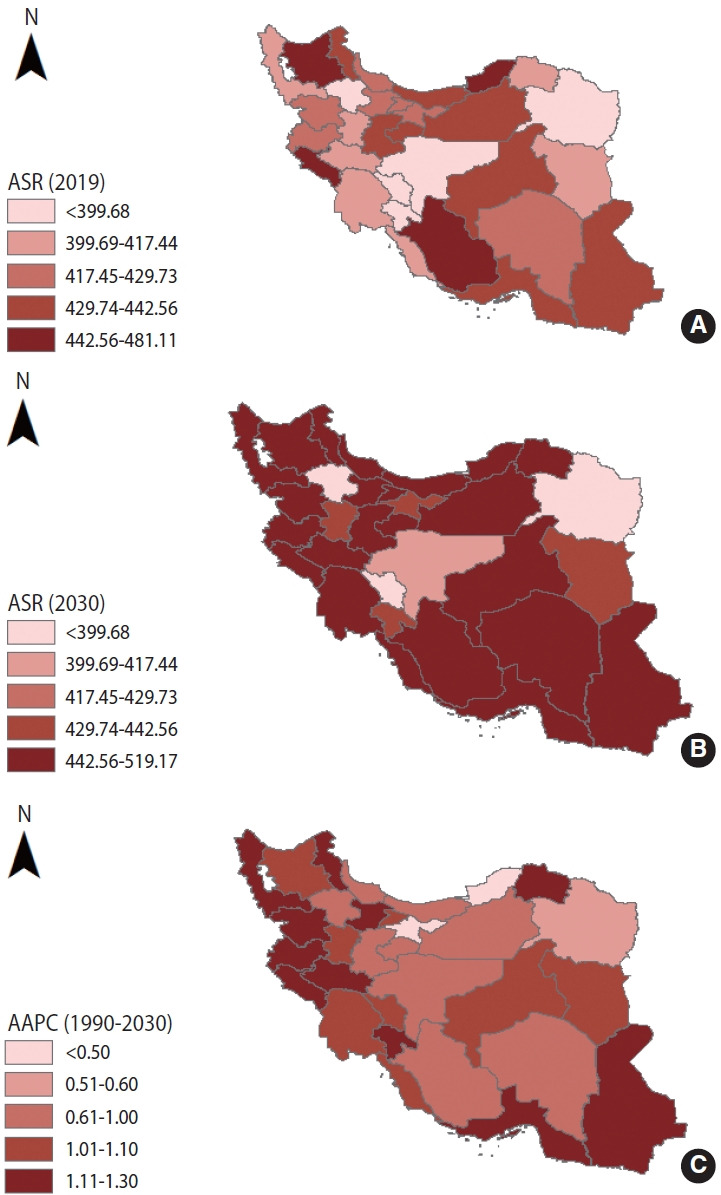
The national distribution and the average annual percentage changes (AAPCs) in the age-standardized incidence rates (ASRs per 100,000) of chronic kidney disease at the national level. (A) ASRs of chronic kidney disease in 2019. (B) ASR of chronic kidney disease in 2030. (C) AAPCs in chronic kidney disease ASRs between 1990 to 2030.

**Figure 5.**
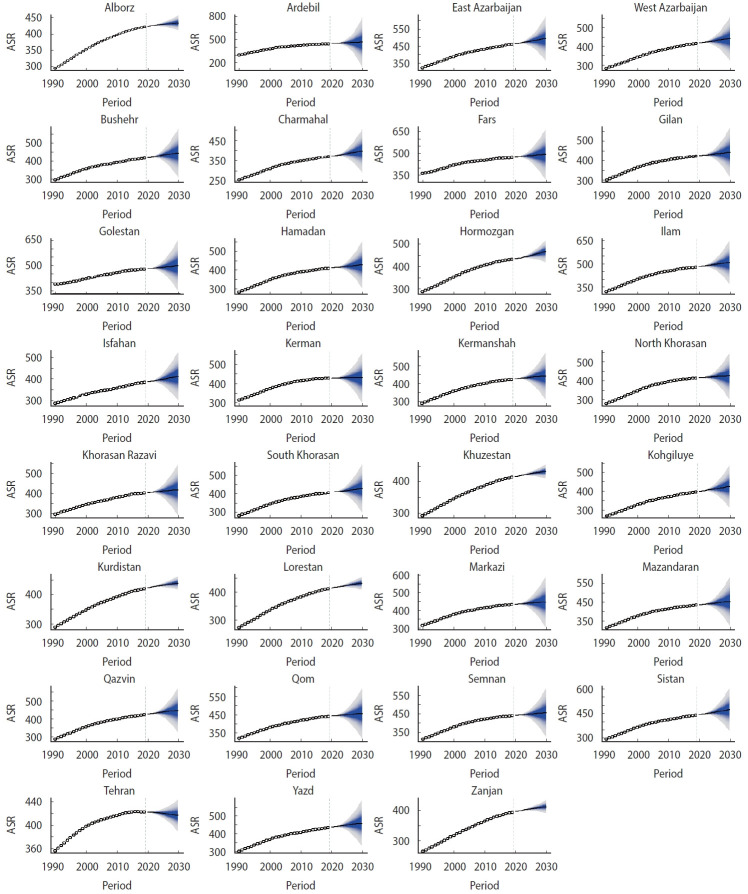
Temporal trends in the age-standardized incidence rates (ASRs) of chronic kidney disease between 1990 and 2019 and their projections through 2030 in all provinces of Iran. The open dots represent the observed values, and the fan shape denotes the predicted distribution between the 2.5% and 97.5% quintiles. The predicted mean value is shown as a solid line. The vertical dashed line indicates where the prediction starts.

From 2020 to 2030, the CKD case number is predicted to increase to 423,300 (Table 2 and Figure 2). The ASR of CKD will increase from 390.87 per 100,000 people to 469.04 per 100,000 people during the same period (AAPC, 1.9%; 95% CI, 1.8 to 2.0) (Table 2 and Figure 2). The ASR in 2030 was approximately 2.5 times that of 1990. Between 2020 and 2030, the CKD case number will increase to 192,300 thousand in male and 236,800 in female. The ASR of CKD will increase to 426.67 per 100,000 in male (95% credible interval [CrI], 370.53 to 482.81) and 512.86 per 100,000 in female (95% CrI, 420.86 to 604.87). An increasing trend in case numbers and ASR is expected for both sexes. The case number is predicted to decrease in the youngest groups (aged under 19) from 2020 to 2030. However, a persistent increase in case numbers is expected for people aged 20-39 years, 40-59 years, and older than 60 years from 2020 to 2030 (Table 2 and Figure 3). The 31 provinces of Iran will experience an increasing CKD ASR between 2020 and 2030. The greatest increase is expected in the Lorestan and Hormozgan Province (AAPC, 1.3%; 95% CI, 1.2 to 1.4), and the smallest increase is expected in Tehran and Golestan Provinces (AAPC, 0.5%; 95% CI, 0.4 to 0.6) (Figure 4). More details about the temporal trend in the ASRs of CKD per 100,000 between 1990 and 2019 and their projections up to 2030 for separate provinces in Iran are depicted in Figure 5.

## DISCUSSION

The incidence of CKD varies widely worldwide. Although the burden attributed to this disease is higher than that of all common cancers, its importance has not received a corresponding level of attention, either globally or regionally. According to our findings, CKD cases and age-specific incidence rates are expected to increase in Iran through 2030, continuing trends from 1990. This increasing trend was observed in both male and female. A consistent decrease in CKD incidence rates and case numbers was found in children and adolescents (age group under 19 years). However, a consistent increase was observed in young, middle-aged, and older adults (age group ≥ 20 years). These increasing trends might soon pose a major challenge to the health system.

It is reasonable to examine CKD risk factors to explain this increasing trend. The role of genetic and environmental factors in the occurrence and progression of CKD has been investigated and documented [20,21]. Smoking increases the risk of CKD through a proinflammatory state, oxidative stress, prothrombotic shift, endothelial dysfunction, glomerulosclerosis, and tubular atrophy [22]. Based on previous research, the risk of CKD in current smokers and past smokers is 30% and 15% higher than in non-smokers, respectively [23]. It is noteworthy that each additional 5 smoked cigarettes per day are associated with an increase in serum creatinine by 31%. With an increase in blood creatinine, the kidneys’ ability to detoxify and clean up waste decreases, thereby increasing the risk of CKD [24]. In Iran, it is estimated that a significant proportion of the general population smokes (19.8% of male, 3.6% of female and 13.9% of the total population), and the trend of tobacco smoking is also increasing [25,26]. Furthermore, obesity and overweight are the strongest modifiable risk factors for CKD in the 21st century, exhibiting a striking increase over the past four decades [23,27]. Obesity leads to kidney damage through inflammation, endothelial dysfunction, oxidative stress, hypervolemia, and a prothrombotic state [28]. Moreover, alcohol consumption has been associated with CKD progression; the global consumption in adults increased from 5.9 L to 6.5 L and is forecasted to reach 7.6 L by 2030 [29]. This alarming increase in smoking, alcohol consumption per capita, and overweight and obesity might drive an unexpected increase in CKD incidence in Iran.

One of the most critical risk factors for CKD is T2DM. According to our findings, CKD due to diabetes mellitus will continue to increase until 2030. Based on the previous research, the national trend of T2DM in Iran is increasing (the prevalence of T2DM increased by 35.1% over a 7-year period), and patients with T2DM have an increased risk of diabetic complications, including CKD [30]. The mechanisms that lead to CKD in diabetes include hyperfiltration injury, advanced glycosylation end products, and reactive oxygen species [31]. At the molecular level, numerous cytokines, growth factors, and hormones such as transforming growth factor-beta and angiotensin 2 cause pathologic changes associated with diabetic nephropathy [23,31]. Meanwhile, the demographic transition to aging in Iran is associated with an increase in T2DM and subsequent CKD due to diabetes [32]. Through these factors, in the absence of new and effective preventive interventions, the increasing global prevalence of T2DM will inevitably be accompanied by an increase in CKD prevalence.

The increasing trend of CKD incidence and new cases in Iran also might be attributed to the increasing prevalence of hypertension. Hypertension is a definite risk factor for CKD that leads to glomerulosclerosis and loss of kidney function [23]. Based on a systematic review and meta-analysis conducted in 2016, hypertension is one of the most common health problems in Iran, and around one-quarter of the adult population has high blood pressure. The prevalence of hypertension increases with age [33]. Therefore, appropriate and timely public health management interventions regarding hypertension are essential for CKD control and prevention.

Another reason for the rising incidence of CKD during the last 3 decades and the future decade in Iran may be due to the shifting lifestyle toward Western diets with a high level of calories and the expansion of a sedentary lifestyle. Based on previous research, participants with a high level of physical activity had a lower risk of developing CKD than inactive participants. In addition, the prevalence of Western diets—and hence, overweight and obesity—may also lead to an increased risk of CKD incidence and progression [23,34,35].

To the best of our knowledge, our study is the first to systematically explore the trends in CKD incidence from 1990 to 2019 using the available information on incidence rates in Iran from the GBD data. We were also able to predict CKD incidence up to 2030, providing evidence for future policy-making. Nonetheless, our study had some limitations. First, the temporal trends of CKD incidence in both the past and the future might be partly influenced by the detection and reporting rates. Underreporting and detection biases are even higher in low- and middle-income countries such as Iran. Second, the GBD data were estimates from mathematical models based on surveillance data rather than reflecting actual surveillance data. Despite these limitations, by using the most up-to-date data and advanced modeling strategies, our study provides a convincing prediction of CKD incidence in the years to come. Considering this last limitation, we compared the values estimated by the GBD query tool with the actual values reported in previous studies in Iran. According to the estimated data of the GBD website, the prevalence of CKD fluctuated between 5.10% and 10.69% between 1990 and 2019. For this purpose, we searched PubMed for the keywords (((Incidence) OR (Prevalence)) AND (((((Renal Insufficiency, Chronic) OR (Chronic Renal Insufficiencies)) OR (Chronic Renal Insufficiency)) OR (Chronic Kidney Disease)) OR (Renal Disease, Chronic))) AND (Iran). Most of the studies published in Iran were related to the prevalence of CKD, rather than the incidence of CKD. This systematic search revealed that the prevalence of CKD in southwestern Iranian children was 6.47% in 2009 [36]. In another study by Madani et al. [37] in 2001, the prevalence of chronic renal failure was 10.24%. In the studies of Shahdadi et al. [38], Sorkhi & Bizhani [39], Khajehdehi et al. [40], Mahdavi-Mazdeh et al. [41], and Najafi et al. [42], the prevalence of CKD was 8.65%, 5.88%, 5.33%, 6.29%, and 5.14%, respectively, which was consistent with the values estimated by the Global Health Data Exchange website. Moreover, based on a systematic review and meta-analysis in 2018, the prevalence of CKD in the Iranian general population 7 years to 86 years old was 8.24% (95% CI for effect size, 5.58 to 10.9), which is consistent with the values estimated by the GBD website [43]. In terms of the incidence rate, we used the study of Nafer et al. [44], which calculated the burden attributed to CKD. Their research indicated that diabetes mellitus, hypertension, and glomerulonephritis accounted for 41.1%, 38.3%, and 16.5% of incident cases of CKD, respectively, which is consistent with the values presented in this study [44]. Regarding comparisons with other countries that have similar environments to Iran, we compared our estimated values with China, Iraq, Syria, and Egypt. These comparison locations were chosen based on socio-demographic indicators. Based on a cross-sectional survey in China, the overall prevalence of CKD was 10.8% (95% CI, 10.2 to 11.3) [45]. In another study in different Chinese areas the prevalence of CKD in Beijing, Guangzhou, and the village of Zhejiang was 11.3%, 10.1%, and 13.5%, respectively [46]. The prevalence of CKD in Iraq, which is Iran’s western neighbor, was 6.8% in a study by Kamil et al. [47] in 2021. In a population screening in Egypt to determine the prevalence of chronic renal insufficiency, the rate was estimated at 10.6% [48]. Likewise, the prevalence of CKD in Syria was 11% in 2005 [49]. A review of previous findings conducted in Iran and different countries with similar environments showed that the estimated values of CKD by the GBD website, which we used in the present study, are consistent with the values presented in studies using real-world data.

The validity and logic of using BAPC models have been discussed previously in detail by Riebler & Held [19]. In summary, unlike methods such as Lee-Carter and Nordpred, which report point forecasts, BAPC has a prediction interval, which is helpful in clinical decisions where we constantly see uncertainty. As there will always be substantial uncertainty in demographic forecasts, these forecasts should be probabilistic. Another positive aspect of the BAPC model is that for predicting further into the future than 10 years, the exponential trend assumed by the BAPC model is helpful. Furthermore, for smaller countries where sparsity is present or datasets contain zero counts, using the BAPC model does not create a problem in the forecast. As another benefit, unlike the forecasts of the World Health Organization for various outcomes, which are presented for 5-year time intervals, the BAPC method makes it possible to develop annual forecasts. In relation to the last point, Riebler & Held [19], in a study published in 2017, noted that this approach is also attractive for topics other than cancer surveillance.

In summary, the CKD case numbers and incidence rates were predicted to increase in the next decade until 2030. The most pronounced increase is predicted among people with T2DM, people with hypertension and older people, suggesting that current prevention strategies should focus on these high-risk populations, which should also be prioritized for future strategies targeting the national control of chronic kidney disease. Effective prevention measures are still needed to alleviate the CKD burden imposed by T2DM and hypertension. The long-term best-practice approach should include the primary prevention of smoking, alcohol consumption, and obesity.

## Figures and Tables

**Figure f6-epih-45-e2023027:**
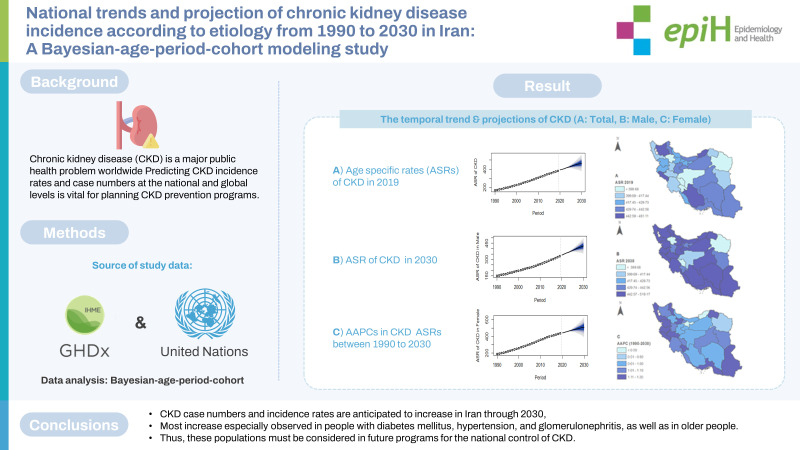


**Table 1.** The number and age-specific incidence rate of CKD by sex, etiology, and age groups from 1990 to 2019 in Iran

Variables		Case, n (×1,000)		ASR (×100,000)		AAPC (95% CI)^1^ of ASR
		1990	2019	1990 (95% CrI)	2019 (95% CrI)	1990-2019
Sex						
	Both	97.3	315.5	168.52 (166.32, 170.71)	382.98 (380.79, 385.18)	2.9 (2.8, 3.0)
	Male	43.7	139.4	149.38 (148.05, 150.71)	330.61 (329.28, 331.94)	2.8 (2.7, 2.9)
	Female	53.5	176.1	188.43 (184.45, 192.42)	436.57 (432.58, 440.55)	2.9 (2.8, 3.0)
Etiology						
	Diabetes mellitus (type 1)	1.4	2.8	2.32 (2.25, 2.38)	3.24 (3.17, 3.30)	1.2 (1.1, 1.3)
	Diabetes mellitus (type 2)	11.7	41.0	20.02 (19.81, 20.23)	49.03 (48.82, 49.24)	3.1 (3.0, 3.2)
	Hypertension	6.6	24.6	11.22 (11.09, 11.34)	29.34 (29.21, 29.46)	3.4 (3.3, 3.5)
	Glomerulonephritis	5.9	11.5	10.21 (10.04, 10.37)	14.06 (13.90, 14.22)	1.1 (1.0, 1.2)
	Other causes	72.1	236.6	124.75 (123.08, 126.43)	287.31 (285.63, 288.98)	2.9 (2.8, 3.0)
Age (yr)^2^						
	0-19	15.1	14.5	45.7 (43.3, 48.0)	60.9 (59.3, 62.6)	1.0 (0.9, 1.1)
	20-39	5.9	19.4	42.2 (40.9, 43.4)	65.7 (63.4, 67.9)	1.5 (1.4, 1.6)
	40-59	28.8	105.1	424.8 (419.1, 430.5)	547.1 (535.8, 558.4)	0.9 (0.8, 1.0)
	≥60	47.3	176.4	1,521.7 (1,507.2, 1,536.3)	2,149.1 (2,131.7, 2,166.6)	1.2 (1.1, 1.3)

CKD, chronic kidney disease; ASR, age-standardized incidence rate; AAPC, average annual percentage change; CrI, credible interval; CI, confidence interval.

1 The 95% CIs of AAPC were calculated using a joinpoint regression model.

2 The incidence rates for age groups have not been standardized by age.

**Table 2.** The predicted case numbers and ASR of CKD in 2020, 2024, and 2030, by sex, etiology, and age groups, estimated using a BAPC model

Variables		Case, n (×1,000)			ASR (×100,000)			AAPC (95% CI)^1^ of ASR	
		2020	2024	2030	2020 (95% CrI)	2024 (95% CrI)	2030 (95% CrI)	2020-2024	2020-2030
Sex									
	Both	325.1	366.1	423.3	390.87 (390.78, 390.97)	422.57 (414.11, 431.04)	469.04 (399.20, 538.87)	2.0 (1.9, 2.1)	1.9 (1.8, 2.0)
	Male	142.8	164.3	192.3	339.51 (339.43, 339.59)	374.58 (368.43, 380.74)	426.67 (370.53, 482.81)	2.6 (2.5, 2.7)	2.4 (2.3, 2.5)
	Female	184.2	202.4	236.8	443.45 (443.35, 443.46)	471.92 (461.58, 482.25)	512.86 (420.86, 604.87)	1.6 (1.5, 1.7)	1.5 (1.4, 1.6)
Etiology									
	Diabetes mellitus (type 1)	2.8	3.0	3.6	3.29 (3.28, 3.30)	3.47 (3.39, 3.56)	3.55 (2.86, 4.23)	1.5 (1.4, 1.6)	1.0 (0.8, 1.1)
	Diabetes mellitus (type 2)	41.7	47.4	55.1	50.19 (50.18, 50.20)	54.85 (53.92, 55.77)	61.85 (54.27, 69.42)	2.3 (2.2, 2.4)	2.2 (2.1, 2.3)
	Hypertension	24.9	28.3	32.6	30.01 (30.00, 30.02)	32.69 (32.14, 33.26)	36.67 (32.06, 41.29)	2.2 (2.1, 2.3)	2.1 (2.0, 2.2)
	Glomerulonephritis	11.9	12.9	14.5	14.22 (14.21, 14.23)	14.88 (14.43, 15.33)	15.76 (12.13, 19.39)	1.1 (1.0, 1.2)	1.1 (1.0, 1.2)
	Other causes	243.9	274.3	317.1	293.16 (293.02, 293.23)	316.69 (310.37, 323.01)	351.12 (298.61, 403.62)	2.0 (1.9, 2.1)	1.9 (1.8, 1.7)
Age (yr)^2^									
	0-19	15.1	15.3	14.7	61.97 (61.62, 62.31)	66.04 (61.86, 70.22)	72.15 (58.53, 85.77)	1.1 (1.0, 1.2)	1.5 (1.3, 1.6)
	20-39	19.9	21.9	24.8	67.58 (67.25, 67.92)	75.11 (71.75, 78.47)	86.39 (75.68, 97.10)	2.6 (2.5, 2.7)	2.5 (2.4, 2.7)
	40-59	109.2	121.8	140.8	543.37 (541.00, 545.74)	528.48 (504.54, 552.42)	506.14 (429.82, 582.46)	-0.2 (-0.3, -0.1)	-0.7 (-0.8, -0.6)
	≥60	181.8	206.6	243.7	2,147.42 (2,135.41, 2,159.44)	2,140.58 (2,051.49, 2,229.66)	2,130.30 (1,860.09, 2,400.50)	-0.3 (-0.4, -0.2)	-0.1 (-0.2, -0.1)

ASR, age-standardized incidence rate; CKD, chronic kidney disease; BAPC, Bayesian age-period-cohort; CrI, credible interval; CI, confidence interval; AAPC, average annual percentage change.

1 The 95% CIs of AAPCs were calculated using a joinpoint regression model.

2 The incidence rates for age groups were not standardized by age.

## SUPPLEMENTARY MATERIALS

Supplementary Material 1. The number and age specific rate (ASR) of chronic kidney disease (CKD) bay sex, etiology, and age groups from 1990 to 2019 in Iran (Generalized Additive Model)

Supplementary Material 2 The predictive number and ASR of CKD in 2020 and 2030, by sex, etiology, and age groups. The case numbers and Age Specific Rate (ASR) of chronic kidney disease in 2020 and 2030 were estimated by Generalized Additive Model

Supplementary Material 3 The number and age specific rate (ASR) of chronic kidney disease (CKD) bay sex, etiology, and age groups from 1990 to 2019 in Iran (Smooth Spline Model)

Supplementary Material 4 The predictive number and ASR of CKD in 2020 and 2030, by sex, etiology, and age groups. The case numbers and Age Specific Rate (ASR) of chronic kidney disease in 2020 and 2030 were estimated by Smooth Spline Model

Supplementary Material 5 The number and age specific rate (ASR) of chronic kidney disease (CKD) bay sex, etiology, and age groups from 1990 to 2019 in Iran (Joinpoint regression model)

Supplementary Material 6 The predictive number and ASR of CKD in 2020 and 2030, by sex, etiology, and age groups. The case numbers and Age Specific Rate (ASR) of chronic kidney disease in 2020 and 2030 were estimated by Joinpoint regression model

Supplementary Material 7 The number and age specific rate (ASR) of chronic kidney disease (CKD) bay sex, etiology, and age groups from 1990 to 2019 in Iran (Poisson regression model)

Supplementary Material 8 The predictive number and ASR of CKD in 2020 and 2030, by sex, etiology, and age groups. The case numbers and Age Specific Rate (ASR) of chronic kidney disease in 2020 and 2030 were estimated by Poisson regression model
