# Supplementary material for: National trends and projection of chronic kidney disease incidence according to etiology from 1990 to 2030 in Iran: a Bayesian age-period-cohort modeling study
Source: Epidemiol Health. 2023 Feb 17;45:e2023027. doi: 10.4178/epih.e2023027 (PMC10482568; doi:10.4178/epih.e2023027)
Supplement: Supplementary Material 1. — The number and age specific rate (ASR) of chronic kidney disease (CKD) bay sex, etiology, and age groups from 1990 to 2019 in Iran (Generalized Additive Model) [file epih-45-e2023027-Supplementary-1.docx]

**Supplementary Material 1.** The number and age specific rate (ASR) of chronic kidney disease (CKD) bay sex, etiology, and age groups from 1990 to 2019 in Iran (Generalized Additive Model)

|  | Case numbers  (×1000) | | ASR (×100,000) | | AAPC ^ǂ^ (95% CI) of ASR |
| --- | --- | --- | --- | --- | --- |
|  | 1990 | 2019 | 1990 (95% CI) | 2019 (95% CI) | 1990-2019 |
| Sex | | | | | |
| Both | 94.98 | 315.95 | 310.2 (307.8, 312.7) | 427.2 (424.9, 429.5) | 1.1 (0.9, 1.2) |
| Male | 42.97 | 137.91 | 270.7 (267.2, 274.2) | 365.4 (363.0, 367.9) | 1.0 (0.9, 1.1) |
| Female | 52.01 | 178.04 | 354.4 (349.2, 359.6) | 482.6 (477.4, 487.8) | 1.1 (1.0, 1.2) |
| Etiology | | | | | |
| Diabetes mellitus I | 1.31 | 2.67 | 10.3 (10.1, 10.5) | 14.5 (14.4, 14.7) | 1.4 (1.2, 1.5) |
| Diabetes mellitus II | 11.27 | 40.45 | 311.2 (311.0, 311.4) | 400.3 (400.1,400.6) | 1.1 (1.0, 1.2) |
| Hypertension | 6.31 | 24.20 | 174.2 (174.1, 174.4) | 239.9 (139.7, 240.0) | 1.1 (1.0, 1.2) |
| Glomerulonephritis | 5.78 | 11.61 | 10.5 (10.4, 10.6) | 13.6 (13.5, 13.7) | 0.9 (0.8, 1.1) |
| Other causes | 70.31 | 237.02 | 231.3 (229.3, 233.3) | 319.9 (318.0, 321.8) | 1.1 (0.9, 1.2) |
| Age groups (yr)^b^ | | | | | |
| 0-19 | 14.68 | 14.82 | 45.85 (44.58, 47.12) | 58.44 (57.17, 59.71) | 0.9 (0.8, 1.0) |
| 20-3 | 5.85 | 19.52 | 39.38 (38.75, 40.00) | 65.04 (64.42, 65.66) | 1.7 (1.6, 1.8) |
| 40-59 | 27.61 | 105.81 | 424.97 (417.59, 432.35) | 554.87 (547.48, 562.25) | 0.9 (0.8, 1.0) |
| ≥60 | 46.85 | 175.81 | 1538.87 (1525.73, 1552.01) | 2141.11 (2127.97, 2154.25) | 1.2 (1.1, 1.3) |

ǂ Average Annual Percent Change (AAPC)

P< 0.05.

^a^ The 95% CIs of AAPC were calculated by using the Joinpoint regression model

^b^ The incidence rates for age groups have not been standardized by age.
