# Supplementary material for: National trends and projection of chronic kidney disease incidence according to etiology from 1990 to 2030 in Iran: a Bayesian age-period-cohort modeling study
Source: Epidemiol Health. 2023 Feb 17;45:e2023027. doi: 10.4178/epih.e2023027 (PMC10482568; doi:10.4178/epih.e2023027)
Supplement: Supplementary Material 3 — The number and age specific rate (ASR) of chronic kidney disease (CKD) bay sex, etiology, and age groups from 1990 to 2019 in Iran (Smooth Spline Model) [file epih-45-e2023027-Supplementary-3.docx]

**Supplementary Material 3.** The number and age specific rate (ASR) of chronic kidney disease (CKD) bay sex, etiology, and age groups from 1990 to 2019 in Iran (Smooth Spline Model)

|  | Case numbers  (×1000) | | ASR (×100,000) | | AAPC ^ǂ^ (95% CI) of ASR |
| --- | --- | --- | --- | --- | --- |
|  | 1990 | 2019 | 1990 (95% CI) | 2019 (95% CI) | 1990-2019 |
| Sex | | | | | |
| Both | 96.44 | 317.53 | 170.56 (168.00, 173.13) | 382.98 (380.42, 385.55) | 2.8 (2.7, 2.9) |
| Male | 42.55 | 139.00 | 148.89 (147.13, 150.66) | 331.71 (329.95, 333.47) | 2.8 (2.7, 2.9) |
| Female | 53.89 | 178.52 | 192.94 (188.38, 197.50) | 435.47 (430.91, 440.03) | 2.9 (2.8, 3.0) |
| Etiology | | | | | |
| Diabetes mellitus I | 1.38 | 2.72 | 2.41 (2.31, 2.52) | 3.28 (3.18, 3.38) | 1.1 (1.0, 1.2) |
| Diabetes mellitus II | 11.31 | 40.71 | 20.08 (19.79, 20.38) | 49.14 (48.85, 49.44) | 3.1 (3.0, 3.2) |
| Hypertension | 6.40 | 24.32 | 11.33 (11.19, 11.48) | 29.33 (29.19, 29.48) | 3.4 (3.3, 3.5) |
| Glomerulonephritis | 5.83 | 11.65 | 10.30 (10.11, 10.48) | 14.03 (13.85, 14.22) | 1.1 (1.0, 1.2) |
| Other causes | 71.51 | 238.12 | 126.43 (124.51, 128.35) | 287.19 (285.27, 289.11) | 2.9 (2.8, 3.0) |
| Age groups (yr)^b^ | | | | | |
| 0-19 | 14.88 | 15.01 | 48.13 (46.68, 49.58) | 58.03 (56.57, 59.48) | 0.7 (0.6, 0.8) |
| 20-3 | 5.81 | 19.47 | 37.98 (37.16, 38.80) | 66.40 (65.58, 67.22) | 1.9 (1.8, 2.0) |
| 40-59 | 28.98 | 105.53 | 425.37 (415.83, 434.90) | 551.61 (542.07, 561.14) | 0.9 (0.8, 1.0) |
| ≥60 | 46.77 | 177.51 | 1530.69 (1502.53, 1558.84) | 2098.44 (2070.29, 2126.60) | 1.1 (1.0, 1.2) |

ǂ Average Annual Percent Change (AAPC)

P< 0.05.

^a^ The 95% CIs of AAPC were calculated by using the Joinpoint regression model

^b^ The incidence rates for age groups have not been standardized by age.
