# Supplementary material for: National trends and projection of chronic kidney disease incidence according to etiology from 1990 to 2030 in Iran: a Bayesian age-period-cohort modeling study
Source: Epidemiol Health. 2023 Feb 17;45:e2023027. doi: 10.4178/epih.e2023027 (PMC10482568; doi:10.4178/epih.e2023027)
Supplement: Supplementary Material 6 — The predictive number and ASR of CKD in 2020 and 2030, by sex, etiology, and age groups. The case numbers and Age Specific Rate (ASR) of chronic kidney disease in 2020 and 2030 were estimated by Joinpoint regression model [file epih-45-e2023027-Supplementary-6.docx]

**Supplementary Material 6.** The predictive number and ASR of CKD in 2020 and 2030, by sex, etiology, and age groups. The case numbers and Age Specific Rate (ASR) of chronic kidney disease in 2020 and 2030 were estimated by Joinpoint regression model

|  | Case numbers  (×1000) | | ASR (×100,000) | | AAPC ^ǂ^ (95% CI) of ASR |
| --- | --- | --- | --- | --- | --- |
|  | 2020 | 2030 | 2020 (95% CrI) | 2030 (95% CrI) | 2020-2030 |
| Sex | | | | | |
| Both | 308.89 | 358.44 | 384.86 (384.81, 384.92) | 461.01 (460.83, 461.19) | 1.8 (1.7, 1.9) |
| Male | 131.43 | 163.14 | 323.37 (323.32, 323.42) | 384.97 (384.81, 385.13) | 1.8 (1.7, 1.9) |
| Female | 177.46 | 222.29 | 447.65 (447.59, 447.71) | 538.48 (538.28, 538.68) | 1.9 (1.8, 2.0) |
| Etiology | | | | | |
| Diabetes mellitus I | 2.49 | 2.98 | 3.04 (3.03, 3.05) | 3.41 (3.40, 3.42) | 1.2 (1.1, 1.3) |
| Diabetes mellitus II | 39.27 | 49.21 | 49.00 (48.99, 49.01) | 59.04 (59.01, 59.06) | 1.9 (1.8, 2.0) |
| Hypertension | 23.65 | 29.82 | 29.56 (29.55, 29.57) | 35.94 (35.93, 35.96) | 2.0 (1.9, 2.1) |
| Glomerulonephritis | 11.36 | 13.41 | 13.92 (13.91, 13.93) | 15.38 (15.37, 15.39) | 1.0 (0.9, 1.1) |
| Other causes | 232.12 | 290.01 | 289.33 (289.29, 289.38) | 347.23 (347.09, 347.62) | 1.8 (1.7, 1.9) |
| Age groups (yr)^b^ | | | | | |
| 0-19 | 13.73 | 13.85 | 58.64 (58.63. 58.66) | 65.21 (65.16, 65.26) | 1.1 (1.0, 1.2) |
| 20-3 | 19.90 | 24.64 | 61.81 (61.79, 61.83) | 68.77 (68.72, 68.82) | 1.1 (1.0, 1.2) |
| 40-59 | 106.16 | 134.51 | 581.25 (581.09, 581.41) | 631.21 (630.69, 631.72) | 0.8 (0.7, 0.9) |
| ≥60 | 169.09 | 212.42 | 2328.54 (2327.95, 2329.12) | 2560.85 (2558.91, 2562.79) | 1.0 (0.9, 1.1) |

ǂ Average Annual Percent Change (AAPC)

P< 0.05.

^a^ The 95% CIs of AAPC were calculated by using the Joinpoint regression model

^b^ The incidence rates for age groups have not been standardized by age.
