# Supplementary material for: National trends and projection of chronic kidney disease incidence according to etiology from 1990 to 2030 in Iran: a Bayesian age-period-cohort modeling study
Source: Epidemiol Health. 2023 Feb 17;45:e2023027. doi: 10.4178/epih.e2023027 (PMC10482568; doi:10.4178/epih.e2023027)
Supplement: Supplementary Material 7 — The number and age specific rate (ASR) of chronic kidney disease (CKD) bay sex, etiology, and age groups from 1990 to 2019 in Iran (Poisson regression model) [file epih-45-e2023027-Supplementary-7.docx]

**Supplementary Material 7.** The number and age specific rate (ASR) of chronic kidney disease (CKD) bay sex, etiology, and age groups from 1990 to 2019 in Iran (Poisson regression model)

|  | Case numbers  (×1000) | | ASR (×100,000) | | AAPC ^ǂ^ (95% CI) of ASR |
| --- | --- | --- | --- | --- | --- |
|  | 1990 | 2019 | 1990 (95% CI) | 2019 (95% CI) | 1990-2019 |
| Sex | | | | | |
| Both | 79.26 | 301.24 | 156.41 (152.38, 160.45) | 377.25 (373.21, 381.28) | 3.1 (3.0, 3.2) |
| Male | 36.28 | 121.91 | 138.58 (134.02, 143.16) | 317.21 (312.64, 321.79) | 2.9 (2.8, 3.0) |
| Female | 42.98 | 172.98 | 175.15 (170.22, 180.09) | 438.56 (433.63, 443.50) | 3.2 (3.1, 3.3) |
| Etiology | | | | | |
| Diabetes mellitus I | 1.02 | 2.44 | 1.91 (1.76, 2.07) | 3.01 (2.85, 3.16) | 1.6 (1.5, 1.7) |
| Diabetes mellitus II | 9.46 | 38.28 | 18.87 (18.43, 19.32) | 47.99 (47.54, 48.44) | 3.3 (3.2, 3.4) |
| Hypertension | 5.13 | 23.03 | 10.42 (10.16, 10.68) | 28.93 (28.67, 29.19) | 3.6 (3.5, 3.7) |
| Glomerulonephritis | 5.21 | 11.16 | 9.53 (9.31, 9.75) | 13.48 (13.55, 13.99) | 1.3 (1.2, 1.4) |
| Other causes | 58.43 | 226.33 | 115.67 (112.67, 118.66) | 283.55 (280.56, 286.54) | 3.1 (3.0, 3.2) |
| Age groups (yr)^b^ | | | | | |
| 0-19 | 13.38 | 13.72 | 38.93 (36.56, 41.29) | 57.99 (55.62, 60.35) | 1.4 (1.3, 1.5) |
| 20-3 | 5.66 | 19.42 | 40.93 (39.44, 42.43) | 61.12 (59.62, 62.61) | 1.4 (1.3, 1.5) |
| 40-59 | 21.10 | 103.32 | 431.38 (423.45, 439.31) | 576.26 (568.32, 584.18) | 1.0 (0.9, 1.1) |
| ≥60 | 39.11 | 164.76 | 1631.60 (1572.13, 1691.08) | 2305.30 (2245.84, 2364.78) | 1.2 (1.1, 1.3) |

ǂ Average Annual Percent Change (AAPC)

P< 0.05.

^a^ The 95% CIs of AAPC were calculated by using the Joinpoint regression model

^b^ The incidence rates for age groups have not been standardized by age.
